# An Innovative Method for Monitoring Food Quality and the Healthfulness of Consumers’ Grocery Purchases

**DOI:** 10.3390/nu9050457

**Published:** 2017-05-05

**Authors:** Le-Thuy T. Tran, Philip J. Brewster, Valliammai Chidambaram, John F. Hurdle

**Affiliations:** Department of Biomedical Informatics, University of Utah School of Medicine, Salt Lake City, UT 84108, USA; ltran@cs.utah.edu (L.-T.T.T.); phil.brewster@utah.edu (P.J.B.); u0758965@utah.edu (V.C.)

**Keywords:** household food environment, informatics, USDA databases, Healthy Eating Index, Dietary Guidelines for Americans, methodology, nutrition assessment

## Abstract

This study presents a method laying the groundwork for systematically monitoring food quality and the healthfulness of consumers’ point-of-sale grocery purchases. The method automates the process of identifying United States Department of Agriculture (USDA) Food Patterns Equivalent Database (FPED) components of grocery food items. The input to the process is the compact abbreviated descriptions of food items that are similar to those appearing on the point-of-sale sales receipts of most food retailers. The FPED components of grocery food items are identified using Natural Language Processing techniques combined with a collection of food concept maps and relationships that are manually built using the USDA Food and Nutrient Database for Dietary Studies, the USDA National Nutrient Database for Standard Reference, the What We Eat In America food categories, and the hierarchical organization of food items used by many grocery stores. We have established the construct validity of the method using data from the National Health and Nutrition Examination Survey, but further evaluation of validity and reliability will require a large-scale reference standard with known grocery food quality measures. Here we evaluate the method’s utility in identifying the FPED components of grocery food items available in a large sample of retail grocery sales data (~190 million transaction records).

## 1. Introduction

Nutrition-related chronic diseases pose a significant burden on healthcare today, both nationally and globally [1,2,3,4]. A healthy diet can help reduce the risk of these diseases [5,6,7,8]. As identified by the Centers for Disease Control and Prevention (CDC) in the United States, common unhealthy diet risk factors linked to chronic diseases include inadequate consumption of fruits/vegetables and excessive intake of sodium and saturated fats [9,10,11]. A healthy diet must therefore consist of a balanced intake of different food groups in the right quantities. In the United States, such a balanced intake is embodied in the Dietary Guidelines for Americans (DGA) developed by the United States Department of Agriculture (USDA) and the United States Department of Health and Human Services [12]. The DGA aims to promote overall health and to reduce the risk of chronic disease, and yet studies show most Americans are unaware of the DGA [13]. Effective use of the DGA for healthy eating in a wider population clearly is desirable to reduce the risk of nutrition-related chronic diseases and ultimately to reduce its burden on the healthcare system. 

Consequently, the DGA is the bedrock of federal nutrition policy and educational programs in the US. It is important to assess the nation’s compliance to these federal guidelines in order to identify and address gaps in the subsequent revised releases of the DGA. In the US, the Healthy Eating Index (HEI) is designed in particular to measure adherence to the DGA. One way to calculate the HEI of individuals/households/populations is to map the individual level dietary data to the USDA’s Food and Nutrient Database for Dietary Studies (FNDDS), which in turn directly links to the USDA’s Food Pattern Equivalents Database (FPED) [14,15,16]. The FPED characterizes different kinds of food and beverages into 37 components that are relevant to the calculation of the HEI. In the United Kingdom, the Nutrient Profiling Model allows foods to be scored and ranked based on recommendations set by the UK Food Standards Agency [17]. Researchers in New Zealand and Australia use the New Zealand Nutrient Profiling Scoring Criterion model to compare food quality at the food item level [18]. 

Rather than focus on population-level or individual-level dietary and food quality assessment, our work focuses on the household level of food acquisition. There are good reasons to focus on the household food environment: (1) this is where most children learn their future eating habits [19,20]; (2) low income households receive benefits, like the Supplemental Nutrition Assistance Program (SNAP) in the US, at the household level; and (3) we have shown that it is possible to make reasonable estimates of food quality based on grocery sales data that necessarily are limited to the household level [21,22,23,24]. The use of grocery sales data avoids recall bias. Additionally, stores routinely collect these data for other purposes and the vast majority of grocers retain these data for months, making the approach potentially quite scalable.

Food purchase or food acquisition data collected at the household level traditionally has been used to track dietary trends in the total population and within population subgroups. For example, in the US, the USDA Economic Research Service’s National Household Food Acquisition and Purchase Survey (FoodAPS) [25] captures household food purchase and acquisition data from a nationally representative sample to inform policy making on key national priorities, including health and obesity, hunger, and nutrition assistance policy. A similar effort in the United Kingdom, the Expenditure and Food Survey (EFS), collects food acquisition data of households to measure food consumption patterns and to estimate the nutrient intake quality of the entire population [26]. Notably, these nationally representative food acquisition surveys collect data only for a few days, which is not sufficient to capture the habitual food consumption patterns of a household over time, especially considering seasonal changes in the retail food market.

The long-term goal of our work is to realize the benefits, described above, of using grocery sales data at scale to assess household grocery food quality. The critical first step of that process relies on robust mapping algorithms that automatically map grocery transaction data to FNDDS food codes. Once FNDDS food codes are identified, the Food Pattern Equivalents Database values that drive the calculation of the HEI can be estimated [23,24]. This mapping process is the focus of this paper. 

## 2. Materials and Methods

Our method is built on an automated model for identifying the FPED component values of grocery market items, starting with the short and concise label descriptions tagged to grocery items, namely, the text that appears on grocery receipts. This is the most generalizable way to process grocery sales data. Not all grocers store long and complete item descriptors for their grocery items, and any given grocer may use its own unique descriptors to describe the same item sold by other grocers. Store brands, foods manufactured for a grocer under a store-specific label, are also captured by this generalized approach. Using our model, we evaluate the results of identifying the FPED components of more than 90,000 distinct grocery items provided by our grocery retail partner.

### 2.1. Grocery Retailers’ Point-of-Sale Transaction Data

For inventory and marketing purposes, most grocery retailers collect detailed point-of-sale transaction data. We received a dataset donation of sales records from a US grocery retail chain containing the shopping activity of 144,000 households in four US geographical regions, spanning a period of 15 months from January 2012 to March 2013. Among other data elements, the sales transaction records contained the Universal Product Code (UPC) or Price Lookup Code (PLU, used mainly for produce, bakery/deli items, and items sold in bulk) of the purchased item, a household identifier, an abbreviated product description, a transaction date/time, and the amount of dollars spent ($US) per item. We identified 92,062 distinct food items in the donated dataset. The food items are organized by the retailer using hierarchical categories with three levels: sub-commodities, commodities, and departments, where sub-commodity is the most granular category. The hierarchical structure of these food items includes 1977 sub-commodities, 327 commodities, and eight departments. Using other online resources, weight or volume information was available for some of the items sold in their original manufactured packaging but is missing for random-weight or re-packaged items such as meat, fruit, and vegetables. This dataset is stored locally in a secure, high-performance computing environment at the University of Utah’s Center for High Performance Computing. Institutional Review Board approval was obtained for this project under University of Utah IRB #18830 (exempt). 

In order to apply a quality measure such as the HEI-2010 at the household level in this dataset, we needed to estimate the Food Pattern Equivalents Database components for all food items purchased by the household over the time window of interest, as well as to estimate the amount (weight) of each food in all transactions. We developed an automated process to link food items to their corresponding Food and Nutrient Database for Dietary Studies food codes in order to pinpoint their FPED components. Linkage used the abbreviated (receipt) product descriptor, the retailer’s hierarchy, and the FPED. The product descriptor for each of the 92,062 food items is a compact, often abbreviated character string. As an illustration, consider the following examples of hypothetical but representative product descriptors: ‘BKRY PRSL100% WW SANW BUNS 11.25 OZ’ for the item ‘Bakery Pre-sliced 100% Whole Wheat Sandwich Buns 11.25 Ounces’; or ‘BK 100% W WHE ENL MUF’ for the item ‘Bakery 100% Whole Wheat English Muffin’; or ‘BKERY 100% WHL WHT WHI BRD' for the item ‘Bakery 100% Whole Wheat White Bread’. Note that a given term in these descriptors may be abbreviated in many different forms (e.g., whole wheat as ‘WHL WHT’ or ‘WW’) and that these abbreviations are often arbitrary. 

### 2.2. USDA’s Food Patterns Equivalents Database (FPED)

The most recent release of the Food Pattern Equivalents Database provides the breakdown of 37 Food Pattern equivalents contained in 100 grams of each of the 8000+ foods and beverages contained in the What We Eat In America (WWEIA) dataset. WWEIA is populated by foods reported in the CDC’s comprehensive National Health and Nutrition Examination Survey (NHANES 2011–2012). For example, the protein food components of FPED include Nuts and Seeds, Eggs, Soybean Products, Meat, Poultry, Cured Meat, Seafood (both low and high omega-3), and Organ Meat. NHANES records food and beverages “as consumed in the America diet”, so we may not always find a good match in WWEIA for our “as purchased” grocery food items. Each FNDDS food code is associated with a full FNDDS food description. This informative text string can be used in our algorithm for finding FPED components of the grocery items, including common components like Fruits, Vegetables, Grains, Proteins, Dairy, Oils, Added Sugars, Solid Fats, and Alcoholic Drinks, as described below. There are 150 What We Eat In America food categories that are used to group together similar FNDDS food codes based on their nutrient content. We augmented WWEIA by adding new categories for whole grain foods. The USDA assigns each food category a four-digit number and description. Each of the FNDDS food codes has been mapped by the USDA to one of the mutually exclusive food categories. 

### 2.3. Food Concept Maps and Relationships

**Figure 1:**
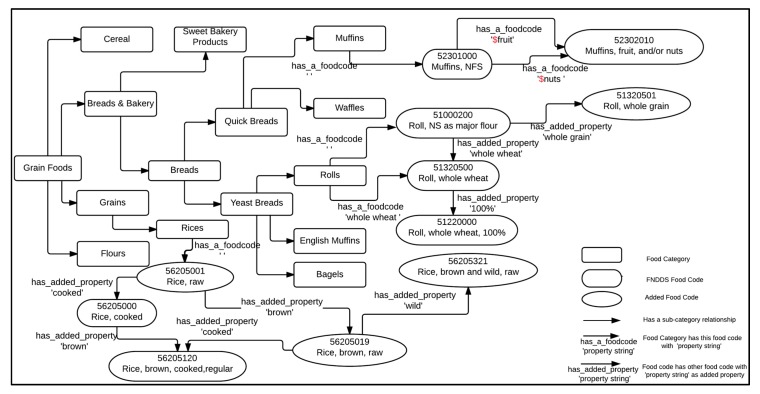
A subset of ‘Grain Foods’ food concepts with their relationships. FNDDS: USDA’s Food and Nutrient Database for Dietary Studies.

We discovered that no single table or database from the USDA contained all the information we needed to map grocery items to the FPED, so we created a custom Food Concept Map. The Map resembles an ontology in that it contains a set of food concepts and key relationships between them. It is based on the information obtained from the FNDDS, the USDA National Nutrient Database for Standard Reference (SR), the WWEIA food categories, and the hierarchical organizations of food items at grocery stores. Figure 1 illustrates a subset of food concepts and their relationships. We consider two different types of food concepts, namely, Food Category and Food Code, which are rendered using different shapes as shown in Figure 1. 

Similar food items are grouped into a distinct concept called a ‘Food Category’ in our concept map (represented by rectangles in Figure 1). A Food Category concept may relate to other Food Category concepts via parent-child relationships. In a parent-child relationship, the child concept represents a sub-category of the parent concept; for example, ‘English Muffins’ is a sub-category of ‘Yeast Breads’. The root concepts at the base for the hierarchical relationships of Food Category concepts are ‘Grain Foods’, ‘Baby Food Products’, ‘Beverages’, ‘Condiments and Sauces’, ‘Dairy Products’, ‘Fruits’, ‘Mixed Dishes’, ‘Meats’, ‘Seafood’, ‘Nuts and Seeds’, ‘Eggs’, ‘Soy Products’, ‘Snacks and Sweets’, ‘Vegetables’, and ‘Ethnic Foods’.

Food Code concepts include the individual food items found in the Food and Nutrient Database for Dietary Studies database. Since retailers fill space with tens of thousands of food UPCs, Food and Nutrient Database for Dietary Studies food codes were insufficient to fairly represent the retail food space. As such, we have included additional nodes in our concept map called the ‘Added Food Codes’ that represent food items in our grocery store dataset that did not have a close match in the FNDDS database. Each Food Code concept contains a food code number, a description, and its Food Pattern Equivalents Database component values. The food code number for the FNDDS food codes are obtained from the FPED directly. We assigned a synthetic food code number to the Added Food Codes using eight-digit numbers not already used in the FPED. The FPED components values for a Food Code concept are the amounts of Food Patterns equivalents contained in 100 g of solid foods or in 100 mL of liquid foods. While the component values for the solid foods from FNDDS Food Code concepts are obtained directly from the FPED, the component values for the liquid foods concepts require conversions to the appropriate values by using estimated densities. In addition, we estimated the FPED component values for the Added Food Code concepts using the conversion techniques from “as purchased” to “as consumed” discussed in [14]. In Figure 1, the concepts 56205001 (Rice, raw) and 56205019 (Rice, brown, raw) are examples of Added Food Code concepts. 

As shown in Figure 1, there are two different types of relationships associated with Food Code concepts: relationships between a Food Category concept and a Food Code concept, and relationships between two different Food Code concepts. Each Food Category concept may have several associated Food Code concepts with different property strings; for example, three Food Code concepts 51000200 (Roll, NS as to major flour), 51320500 (Roll, whole wheat), and 51320501 (Roll, whole grain) relate to the Food Category concept ‘Rolls’ via three property strings ‘, ‘whole wheat’, and ‘whole grain’, respectively. Also, as shown in Figure 1, a Food Code concept may relate to other Food Code concepts via property strings; for example, the Food Code concept 56205120 (Rice, brown, cooked, regular) relates to two different Food Code concepts 56205000 (Rice, cooked) and 56205019 (Rice, brown, raw) with the property strings ‘brown’ and ‘cooked’.

As shown on the top right corner of Figure 1, we add the ‘$’ sign to the property strings ‘fruit’ and ‘nuts’ to indicate that these are our defined categorical variables. Some examples of these defined categorical variables include ‘$fruit’ taking on the values {‘fruit’, ‘strawberry’, ‘banana’, ‘blueberry’, ‘mango’, …}; ‘$nuts’ taking on the values {‘nuts’, ‘almonds’, ‘peanut’, …}, and ‘$tropicalfruit’ taking on the values of {‘tropical fruits’, ‘mango’, ‘pineapples’, …}.

### 2.4. Similarity Metrics to Quantify the Match between Abbreviated Grocery Descriptions and Food Category Concepts

The semantic engine of our framework computes the similarity between the grocery descriptor and the food categories in the concept map based on: the lengths of the two strings; the number of space-separated tokens; and the total number of characters in the strings. A token here represents a sequence of characters separated by space. The string ‘Rice brown raw’, for example, contains three space-separated tokens. Say we want to disambiguate the abbreviated text product description ‘BK 100% W WHE ENL MUF’. We developed an algorithm to extract parts of the abbreviated text that may represent a term in the full description from FNDDS. In this case, our algorithm was able to extract the part ‘W WHE’ as the abbreviation for the term ‘Whole Wheat’. The algorithm is designed to handle what are known as overloaded terms, where a single grocery abbreviation may represent multiple descriptive Food Concept Map terms. The algorithm tries to find the best possible match. We defined a confidence coefficient of a possible match between a full description term FTerm and an extracted abbreviated term AbbTerm by the ratio of their string lengths as follows:

$$CCoef\left(FTerm,\;AbbTerm\right)=\frac{StringLength\left(AbbTerm\right)}{StringLength\left(FTerm\right)}$$

Applied to the above example, we have the confidence coefficient between a full description and an abbreviated string $CCoefMatch\left(\prime Whole\;Wheat^{\prime },\prime W\;WHE\prime \right)\sim 0.45.\;$We consider a full description to be a possible match to an abbreviated string only if the confidence coefficient is equal or greater than 0.4, a value we derived empirically. 

We compiled a list of full description terms, along with their equivalences, to handle the cases of synonyms, acronyms, and other variations. For example, in this list, the full description term ‘Whole Wheat’ has the following equivalences: ‘WholeWheat’, ‘WW’, and ‘W W’; the full description term ‘Fat Free’ has the following equivalences: ‘FatFree’, ‘Nonfat’, ‘NF’, ‘FF’, ‘Non Fat’, ‘Skim’, and ‘0%’; and the full description term ‘English Muffins’ has the following equivalences: ‘EnglishMuffin’, ‘English Muffin’, and ‘EnglishMuffins’. As given by the above equation and the list of full description terms’ equivalences, the confidence coefficient CCoefMatch between the full description term ‘Whole Wheat’ and the two different abbreviated strings ‘BK 100% WW ENL MUF’ and ‘BK 100% WHOWHEA ENL MUF’ are 1.0 and 0.7, respectively. 

The number of words and the number of characters of full description terms are also useful when deciding whether a match is the best possible match. We define WordCount(FTerm) and CharacterCount(FTerm) as the number of words and the number of characters in the full description term, respectively. By these definitions, we have WordCount(′Whole Wheat′) = 2 and CharacterCount(′Whole Wheat′) = 11.

### 2.5. Method for Estimating Food Pattern Components of Grocery Items

The task of estimating the Food Pattern Equivalents Database Components for a given grocery food item is equivalent to identifying a Food Code concept within our Food Concepts Map that best represents it and then using that match’s FPED values. We accomplish this with the following steps:

**Step 1:** We manually mapped most of the 327 commodity level descriptions from our grocery transaction dataset to one of the root concepts in the Concept Maps and Relations (only a few commodities had sub-commodities applied to different root concepts). 

**Step 2:** For any given grocery food item, our system traverses the Food Category concepts using a top-down approach and traces the parent-child relationships to find the list of similar Food Category concepts. Using the similarity metric described above, the system computes the confidence coefficient between the grocery food item compact descriptor and every Food Category concept descriptor under each parent node that it lands on. If a possible match is found (CCoefMatch≥0.4), the traversed Food Category concept, FCatConcept, is added to the list of food category possible matches. The system then eliminates from the list the parent concept of any concepts not on the current traversal path. 

**Step 3:** Having exhausted all the Food Category nodes in the map, the method uses the filtered set of Food Categories, FCatConcepts, to find the best possible Food Code match. For each of these Food Category concepts, the method first identifies the list of all related Food Code concepts and their relationships. Using our similarity metric, we then find possible matches between the property strings of these relationships and the grocery item description. The Food Concept Map and its Relationships are structured so that related food codes are interconnected. In other words, Food Codes are a web of concepts where all related concepts will have a formal relationship defined. Instead of stopping at the first matched Food Code concept, our algorithm uses a greedy approach that computes the confidence coefficient for all related Food Code concepts. The system not only takes these concepts into account, but also the formal relationships defined between them. If there is only one Food Code concept in the list of possible matches, it is then selected to be the Food Code concept that best maps the given grocery food item corresponding to the Food Category concept FCatConcept mentioned at the beginning of **Step 3**. If there is more than one Food Code concept with a confidence coefficient of 0.4 or more, the best possible match is chosen based on the following heuristics:

1. Maximize the value of $\underset{i\in Rels}{\sum }WordCount\left(PropString_{i}\right)$ where Rels is the set of all relationships to traverse from the Food Category concept FCatConcept to the Food Code concept FCConcept during the mapping process and $PropString_{i}$ is the property string of the traversed relationships.
2. Maximize the summed confidence coefficient SumCCoef of possible matches between the Food Code concept FCConcept and the grocery food item’s description AbbString which is defined as follows:
  $$SumCCoef\left(FCConcept,AbbString\right)=\frac{\sum_{i\in Rels}StringLength\left(AbbPTerm_{i}\right)}{\sum_{i\in Rels}StringLength\left(PropString_{i}\right)}$$
  where $AbbPTerm_{i}$ is the abbreviated term of $PropString_{i}$ as it appeared inside the string AbbString.
3. Maximize the value of $\underset{i\in Rels}{\sum }CharacterCount\left(PropString_{i}\right)$

**Step 4:** Select one from the list of Food Code concepts that best maps the given grocery food item of different Food Category concepts to be the best match Food Code concept. We choose the best match Food Code concept among the list based on the satisfaction of the following conditions and the subsequent conditions are considered only if there are multiple items satisfying their preceding conditions:

1. Maximize the value of $SumWordCount=\;WordCount\left(FCatDesc\right)+\;\underset{i\in Rels}{\sum }WordCount\left(PropString_{i}\right)$ where Rels is the set of all relationships to traverse from the Food Category concept FCatConcept to the Food Code concept FCConcept during the mapping process, $PropString_{i}$ is the property string of the traversed relationships, and FCatDesc is the description of the Food Category concept FCatConcept.
2. Maximize the overall confidence coefficient AllCCoef of possible matches between the Food Code concept FCConcept of the Food Category concept FCatConcept and the grocery food item’s description AbbString, which is defined as follows:
  $$\begin{array}{l} AllCCoef\left(FCatConcept,FCConcept,AbbString\right)\\ \;=\frac{StringLength\left(AbbFCatTerm\right)+\;\sum_{i\in Rels}StringLength\left(AbbPTerm_{i}\right)}{StringLength\left(FCatDesc\right)+\sum_{i\in Rels}StringLength\left(PropString_{i}\right)} \end{array}$$
  where $AbbPTerm_{i}$ is the abbreviated term of the property string $PropString_{i}$ as it appears inside the string AbbString, and AbbFCatTerm is the abbreviated term of the description FCatDesc as it appeared inside the string AbbString.
3. Maximize the total character count as defined as follows:
  $$SumCharacterCount=\;CharacterCount\left(FCatDesc\right)+\sum_{i\in Rels}CharacterCount\left(PropString_{i}\right)$$

The following is a demonstration using the above method to find the FPED Components of a grocery food item such as ‘LITE PK & BF BOLGNA LUNCH MT’:

**Step 1:** Assigned Food Category concept is ‘Protein Foods’ based on the grocery commodity.

**Figure 2:**
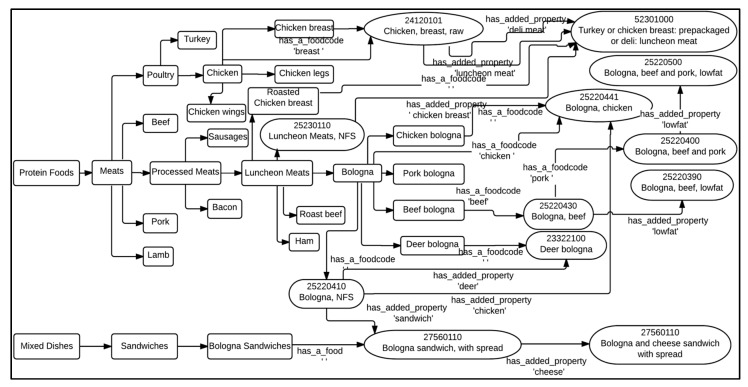
A subset of ‘Protein Foods’ food concepts with their relationships. Relationship arrows without labels are parent-child, “is_a” relationships. Refer to the legend at the lower right corner of Figure 1 for the description of shapes and arrows used here.

**Step 2:** Using all of the nodes that fall under the root node ‘Protein Foods’, as shown in Figure 2 below, the system comes up with a list of possible food category matches. Note that Figure 2 is a snapshot of a small instance of the map and does not show the exhaustive list of child nodes at each level. Table 1 lists all relevant Food Category concepts that have a confidence coefficient ≥0.4. However, the Food Category concepts ‘Bologna’, ‘Luncheon Meats’, and ‘Meats’ are eliminated from the list since they are parent concepts of ‘Beef bologna’, ‘Bologna’, and ‘Beef’, respectively.

**Step 3:** The filtered Food Category concepts and the corresponding Food Code concepts that best map to the given grocery food item are shown in Table 2. There are three Food Code concepts that are best matches of the item corresponding to the three different Food Category concepts: ‘Beef bologna’, ‘Beef’, and ‘Pork’.

**Step 4:** Based on the values of column SumWordCount of Table 2, the Food Code concept 25220500 (Bologna, beef and pork, lowfat) best represents the grocery food item ‘LITE PK & BF BOLOGNA LUNCH MT’.

Note: in the dataset that we received, there are mixed dishes and single-ingredient food items that are being grouped into the same commodity; for example, seafood mixed dishes and single-ingredient seafood dishes are placed in the same commodities. The relationships between the Food Code concepts of ‘Mixed Dishes’ and the Food Code concepts of other root concepts (see Figure 2) allow the above method to find the FPED components of grocery items to work with mixed dishes.

## 3. Results

We applied our method in mapping 90,000+ grocery food items to the FNDDS. The number of food items assigned into each root concept is shown in Table 3. Note that there are concepts which may have different root concepts, such as the mixed dish food items. We also include in Table 3 the number of Added Food Code concepts under each of the root concepts.

A food item is successfully mapped if the method can identify its mapped Food Code concept. We manually reviewed the map to decide if it was an appropriate map for use or not. The number of successful mappings and appropriate mappings corresponding to each of the root concepts is shown in Table 3. We are still in the process of reviewing the mappings for the very complicated set of Mixed Dishes and Ethnic Foods. 

The results in Table 3 show that the method performs very well with the number of successful mappings and the number of appropriate mappings. In the last column of Table 3, we show the percentages of the appropriate mappings over the total number of items being mapped. Once the FPED components of these food items are obtained from USDA tables, the calculation of Healthy Eating Index scores for customers’ household grocery purchases is a viable next step, given an accurate estimation of the food amounts. 

## 4. Discussion

Assessing the healthfulness of consumers’ grocery purchases using household grocery transaction data imposes minimal participant burden, scales gracefully, and is free from recall bias. Although it is not equivalent to monitoring food consumption by individuals, monitoring grocery food purchases can serve as a proxy for the overall quality of the household food environment. As such, it may prove useful in clinical and public health surveillance applications, for example, enabling longitudinal studies to assess the effects of a changing food environment or of educational interventions on diet-related health outcomes. The major contribution of this paper is the development and the implementation of a method for automatically identifying the FPED components of grocery food items that can then be used to estimate household market basket HEI-2010 food component scores over arbitrary shopping intervals. The same information could also be used with other household-level assessment methods that rely on the FPED.

Our work focuses on the household food environment. As we noted in the introduction, there are good reasons to do so (e.g., there is evidence that children learn future eating habits here [19,20]; in many countries food-support subsidies are targeted at the household, not individuals; and our previous work establishes the potential of estimating food quality based on grocery sales at the household level [21,22,23,24]). Focusing on grocery sales holds promise because these data avoid recall bias and because the ubiquity of their collection and storage suggests a high potential for scalability.

Food purchase or food acquisition data collected at the household level traditionally has been used to track dietary trends in the total population and within population subgroups. For example, in the US, the USDA Economic Research Service’s National Household Food Acquisition and Purchase Survey (FoodAPS) [25] captures household food purchase and acquisition data from a nationally representative sample to inform policy making on key national priorities. A similar effort in the United Kingdom, the Expenditure and Food Survey (EFS), collects food acquisition data of households to measure food consumption patterns and to estimate the nutrient intake quality of the entire population [26]. 

When we started this work, we spent a great deal of time trying to identify sources of full-length text descriptions of foods represented by UPCs. As we have discovered since, the use of abbreviated descriptions actually confers an advantage in some cases, because it eliminates excess information that is often included in a full UPC description. Lengthy UPC product descriptions may actually decrease accuracy in the mapping process. For example, the detailed UPC description ‘Pasteurized Skim Milk, Cheese Culture, Salt, Enzymes, Water, Whey, Milk Protein Concentrate, Buttermilk, Sodium Citrate, Sugar, Maltodextrin, Artificial Flavor, Sorbic Acid (Preservative), Carrageenan’ is an actual full description given to a food item with a short grocery description of ‘FF SWISS CHEESE’. Each component of the long descriptor adds a trivial amount of fat when mapped to FPED. It would have been challenging to map the ‘verbose’ full description to the appropriate food code of 14109040 (‘Cheese, Swiss, nonfat or fat free’), owing to the excessive noise contained in the full description. Although this may change with the release of the USDA Branded Food Product Database, it is currently difficult to find free, publicly available full-text UPC descriptors. In the case of our algorithm, it turns out there is less need to do so. Here, less is more.

Future evaluation is needed to confirm if the method presented in this paper is able to facilitate a valid and reliable approach for monitoring household grocery market basket quality over time. Such an evaluation is intrinsically difficult, however, since there is no reference standard at the household level for comparison except the USDA FoodAPS data [25], but that only covers a week’s worth of shopping data. 

## 5. Conclusions

In this study, we presented a method for systematically identifying an essential component required to estimate grocery food quality as expressed by the HEI-2010 using point-of-sale grocery purchase data: an automated process of mapping grocery sales data, in bulk, to FNDDS food codes and thus identifying FPED food components of grocery foods. Once coupled with estimates of food item weights, calculation of grocery food FPED values and HEI-2010 component scores is straightforward. The input to the process is the compact abbreviated descriptions of food items that are similar to those appearing on the point-of-sale receipts of most food retailers. The evaluation in this paper shows the appropriateness of our method for automatically identifying the FPED components of purchased foods. This method may prove useful for household-level food quality dietary assessment. Future evaluation is needed to confirm the utility of the approach at scale and across retail grocery environments.

## Figures and Tables

**Table 1:** Mapping food item ‘LITE PK & BF BOLGNA LUNCH MT’—Finding the possible matches for food category.

Food Category Concepts	FTerm	AbbTerm	CCoefMatch (FTerm,AbbString)
‘Beef bologna’	‘Beef bologna’	‘BF BOLGNA’	0.75
‘Bologna’	‘Bologna’	‘BOLGNA’	0.86
‘Luncheon Meats’	‘Luncheon Meat’	‘LUNCH MT’	0.62
‘Beef’	‘Beef’	‘BF’	0.5
‘Pork’	‘Pork’	‘PK’	0.5
‘Meats’	‘Meat’	‘MT’	1.0

**Table 2:** Mapping food item ‘LITE PK & BF BOLGNA’–Finding the best matches for food codes and the related data.

Possible Representative Food Category Concepts	Best Possible Match Food Code Concepts	Relationship Strings (AbbTerm: CCoefMatch)	SumWordCount
‘Beef bologna’	25220500 (Bologna, beef and pork, lowfat)	‘pork’ (‘PK’:0.5), ‘lowfat’ (‘LITE’:1.0)	7
‘Beef’	21000101 (Beef, NS as to cut, raw)		1
‘Pork’	22000101 (Pork, NS as to cut, raw)		1

**Table 3:** Applied method for identifying Food Patterns Equivalent Database (FPED) components of grocery food items.

Root Concepts	Number of Added Food Code Concepts	Number of Food Items Assigned	Number of Successful Mappings	Number of Appropriate Mappings	Percentage of Appropriate Mappings
Grain Foods	45	5415	4861	4578	85%
Dairy Products	25	5104	4985	4604	90%
Meats	42	12,021	11,590	10891	91%
Seafood	32	3404	3321	3190	94%
Nuts and Seeds	11	865	849	811	96%
Eggs	0	113	113	113	100%
Soy Products	8	226	220	210	89%
Snacks and Sweet	29	15613	15,032	14,127	90%
Fruits	21	2144	2102	1923	89%
Vegetables	18	4099	3923	3878	97%
Beverages	10	11,812	11,224	10,563	89%
Mixed Dishes	38	6792	5203	Review in progress	Not applicable
Condiments and Sauces	35	7482	6192	5789	77%
Baby Food Products	5	732	720	712	97%
Ethnic Foods	22	13,105	11,212	Review in progress	Not applicable
